# An impacted coin in the esophagus: a cause of dysphagia in a child

**DOI:** 10.11604/pamj.2021.39.269.31005

**Published:** 2021-08-25

**Authors:** Alphonce Nsabi Simbila

**Affiliations:** 1 Department of Emergency Medicine, Muhimbili National Hospital, Dar Es Salaam, Tanzania

**Keywords:** esophageal foreign body, dysphagia, child

## Image in medicine

A 4-year-old female with no previous medical history presented to the Emergency Department complaining of difficulty in swallowing especially solid food for a duration of 4 days. This was accompanied by some painful swallowing whenever she attempted taking solid foods. She preferred taking liquids in all the four days. She had no cough, difficulty in breathing, chest pain, stridor, drooling or fever. Physical examination revealed relatively normal oral, neck, abdominal, respiratory and neurological examination. An anteroposterior chest X-rays (A) revealed a round opaque object and a lateral neck X-ray (B) showed a thin opaque object, presenting in a coronal plane, on the same level of the child’s esophagus. Our patient was taken to the emergency operating theatre for endoscopic removal of an esophageal foreign body under anesthesia. A coin was removed without any complications. Initial use of X-ray investigations is important for children with dysphagia before opting for invasive procedures. A coin in the esophagus lies in the coronal view, whereas when situated in the trachea it lies in the sagittal view.

**Figure 1:**
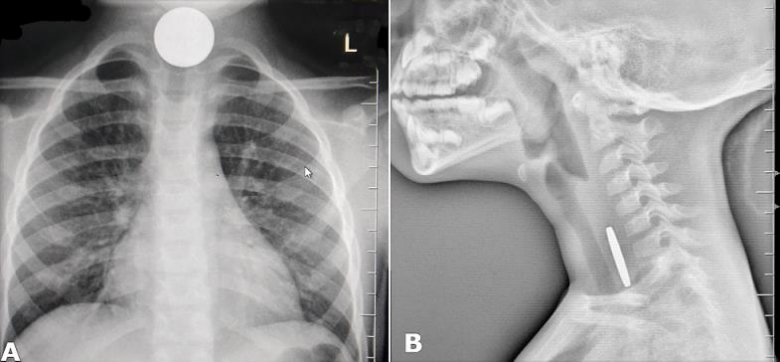
an impacted coin in the esophagus, AP (A), and Lateral (B) views of plain X-rays of the neck

